# Effect of 70% Ethanol Extract and its Solvent Fractions of *Artemisia afra* (Jacq. Ex Willd.) against Pentylenetetrazole-Induced Seizure in Mice

**DOI:** 10.1155/2021/6690965

**Published:** 2021-06-17

**Authors:** Teketel Eristu Kediso, Tesfaye Tolessa, Fikirte Getachew, Eyasu Makonnen, Daniel Seifu

**Affiliations:** ^1^Department of Biomedical Sciences, College of Health Sciences, Arbaminch University, Arbaminch, Ethiopia; ^2^Department of Physiology, College of Health Sciences, Addis Ababa University, Addis Ababa, Ethiopia; ^3^Department of Biomedical Sciences, College of Health Sciences, Wachemo University, Hosanna, Ethiopia; ^4^Department of Pharmacology and Clinical Pharmacy, College of Health Sciences Addis Ababa University, Addis Ababa, Ethiopia; ^5^Center for Innovative Drug Development and Therapeutic Trials for Africa, College of Health Sciences, Addis Ababa University, Addis Ababa, Ethiopia; ^6^Department of Biochemistry, College of Health Sciences, Addis Ababa University, Addis Ababa, Ethiopia; ^7^Division of Biomedical Sciences, Department of Biochemistry, University of Global Health Equity, Kigali, Rwanda

## Abstract

**Introduction:**

*Artemisia afra* (Jacq. ex Willd.), commonly called African wormwood, is a highly aromatic perennial herb and a well-known medicinal plant, claimed to be effective and safe in the treatment of epilepsy. The whole-plant extract is traditionally used as an antiepileptic agent in Ethiopia. *Aim of the Study*. The aim of this study was, therefore, to evaluate the anticonvulsant effect of the hydroethanolic extract and solvent fractions of *A. afra* whole part in mice.

**Materials and Methods:**

The effects of *A. afra* hydroethanolic extract and its solvent fractions were evaluated against pentylenetetrazole- (PTZ-) induced convulsions in mice. The onset and duration of PTZ-induced convulsions were determined with hydroethanolic *A. afra* extract and its solvent fractions. Data were analyzed using a one-way analysis of variance (ANOVA) followed by post hoc Tukey's multiple comparisons test. *p* < 0.05 was considered statistically significant.

**Results:**

The hydroethanolic extract of *A. afra,* with all the three doses of 250, 500, and 1000 mg/kg, showed a significant delay (504.833 ± 62.835^*∗*^ s; *p* < 0.05^*∗*^; 551.833 ± 47.69^*∗∗*^ s; *p* < 0.01^*∗∗*^; and 808.333 ± 64.8^*∗∗∗*^s; *p* < 0.001^*∗∗∗*^, respectively) in the mean onset of convulsion and a decrease (17.000 ± 1.88^*∗∗∗*^ s, *p* < 0.05^*∗*^; 13.000 ± 1.8^*∗∗*^ s, *p* < 0.01^*∗∗*^; and 7.833 ± 1.07^*∗∗∗*^ s, *p* < 0.001, respectively) in the mean duration of convulsion against PTZ-induced convulsion in a dose-dependent manner compared to the control (92.833 ± 13.006 s; 34.167 ± 3.683 s), and its anticonvulsant activity was significantly less compared to that of diazepam (1001.167 ± 68.430 s; 4.500 ± 0.619 s). The solvent fractions, however, did not show anticonvulsant activity against PTZ-induced convulsion.

**Conclusion:**

Crude extract of *A. afra* has an anticonvulsant effect in mice. This might be attributed to the synergistic effects of two or more active ingredients present in the herb.

## 1. Introduction

A seizure is a high-frequency paroxysmal abnormal discharge from an aggregate of neurons in the cerebral cortex, which can be “nonepileptic” when evoked in an ordinary cerebrum by treatment (such as electric shock or chemical convulsant) or “epileptic” when occurring without evident provocation [1].

Epilepsy is a common neurological abnormality characterized by an unpredictable and periodic occurrence of a transient alteration of behavior including convulsions resulting from disordered synchronous and rhythmic firing of brain neurons [2]. It affects about 1% of the world population with an estimated frequency of 50 per 100,000 and 100 per 100,000 in technologically advanced and developing nations, respectively [3].

Modern drug therapy of epilepsy is complicated by the inability of drugs to control seizure in some patients and by their adverse effects ranging from minimal impairment of the central nervous system to death due to aplastic anemia or hepatic failure. Thus, effective and safe therapy of epilepsy remains a challenge [4].

Approximately 30% of the patients continue to have seizures with current antiepileptic drug therapy [5]. Cultural attitudes, lack of prioritization of epilepsy as a public health condition, poor wellbeing framework foundation, and lack of supply of antiepileptic drug plot to upset suitable treatment [6, 7]. The point of treating an epileptic patient is not only to eliminate the event of seizures but also to lead a self-sustained life [5]. Hence, there is a need to look for more effective and safer alternative antiepileptic agents.

Traditional medicine continues to serve a large segment of the population regardless of the advent of modern medicine. The efficacy and safety of these remedies, however, have to be demonstrated through scientific evaluation to prove their therapeutic importance [8]. Previous studies have shown that some of the medicinal plants traditionally used for the treatment of epilepsy possessed promising anticonvulsant activities in animal models, suggesting their potential as sources of newer antiepileptic drugs [9–11].

*Artemisia afra* (family: Asteraceae) named “African wormwood”, also locally known as “Ariti” in Ethiopia, is one of the several claimed herbal medicines used for the treatment of epilepsy [12]. Its other uses include cough, fever, colic, headache, intestinal parasitic diseases, and malaria treatment. Its roots, stems, and leaves are used for the treatments in the form of enemas, poultices, infusions, lotion, or inhalations [13, 14]. The plant grows widely in Ethiopia, specifically in Bale, Oromia Region [12]. Consequently, this study was framed to investigate the antiepileptic effects of the hydroethanolic extract and solvent fractions of *A. afra*, in mouse models.

## 2. Materials and Methods

### 2.1. Plant Materials Preparation

The entire piece of *A. afra* (family: Asteraceae) was collected from Bale National Park, Ethiopia, 550 km away from Addis Ababa, and transported in a dark plastic bag to avoid decomposition by light. It was authenticated and given a voucher number (AAU-NH 02T) at the National Herbarium Museum of Ethiopia, and the plant specimen was deposited for future reference at the National Herbarium, College of Computational and Natural Sciences, Addis Ababa University. The whole fresh plant parts were dried at room temperature under the shade and then powdered using a pestle and mortar. A total of 800 g dried powder was macerated in 70% v/v of ethanol for three days, continuously stirred using an orbital shaker at 120 rpm. The extract was then filtered using gauze (0.1 mm 2 mesh) and Whatman filter paper (size 15 cm) (Whatman® England), and the filtrate was kept in a deep freezer at −27°C and then lyophilized for a week (−52°C, 133 × 10–3 mbar). A total yield of 96.08 g crude extract was obtained and kept in a tightly closed aluminum foil in a desiccator until used.

The crude extract of *A. afra* was further fractionated using petroleum ether, dichloromethane, ethanol, and water as solvents in order to decrease polarity.

Thirty grams of a hydroethanolic extract of *A. afra* was dissolved in a separatory funnel in 100 ml of distilled water. The dissolved extract was partitioned with 3 × 150 ml of petroleum ether at 40–60°C, and the partition was concentrated using a rota-vapor to yield 2.32 g of petroleum ether fraction. The aqueous residue was then partitioned with 3 × 150 ml of dichloromethane, and the filtrate was evaporated to yield 3.78 g of dichloromethane fraction. The remaining aqueous residue was further partitioned with 3 × 100 ml ethanol, and the fractionate was concentrated to yield 4.85 g ethanol fraction. The leftover aqueous residue was then dried using a lyophilizer to yield 9.56 g aqueous residue. The fractions were then kept in a separate tightly closed container with aluminum foil in a desiccator until used.

### 2.2. Drugs/Chemicals/Test Substances Preparation and Administration

Pentylenetetrazol (Sigma Chemical Co., Sweden), diazepam (Medifarma Pharmaceuticals), and sodium chloride (BDH, England) were used in this study. All chemicals were prepared freshly just before use. The organic solvent dimethyl sulfoxide (DMSO), ethanol, dichloromethane, and petroleum ether (Germany) were used in the study. PTZ and diazepam were dissolved in saline solution (0.9% w/v). The crude extract and its fractions were dissolved in distilled water and DMSO (2 : 1 v/v) mixture. Drug solutions were prepared according to the supplier's instructions.

The maximum volume of freshly prepared herbal medicine administered at once in rodents was 1 ml/100 g body weight according to OECD guidelines [15]. The starting dose of *A. afra* crude extract to be administered was 250 mg/kg body weight, and 1/10^th^ of LD_50_ was considered as a middle dose, based on the previous study [13, 16].

### 2.3. Experimental Procedure

Male BALB/c mice weighing between 22 and 30 g were obtained from Ethiopian Public Health Institute, Addis Ababa, Ethiopia, and housed at room temperature under standard nutritional and environmental conditions with relative moisture of 30–70% and 12-hour light-dark cycle. They all had access to water and food *ad libitum* and were denied food 12 h before experimentation. The animals were arbitrarily assigned to 9 treatment groups consisting of 6 mice each.

Group I received the vehicle (normal saline (10 ml/kg) p.o.) and served as a negative control; group II received diazepam (2 mg/kg, p.o.), the standard drug, and served as a positive control; groups III, IV, and V received three different doses (250, 500, and 1000 mg/kg) of the crude extract; and groups VI–IX received 1000 mg/kg of the four solvent fractions each (petroleum ether, dichloromethane, ethanol, and aqueous), 30 min before subcutaneous injection of PTZ (80 mg/kg) into the loose skin fold on the back of the neck of the animals. After PTZ administration, mice were placed in a testing chamber and closely observed for the onset of convulsions and the duration of convulsion for 30 min. Delayed onset and decreased duration of convulsions indicate the ability of the substance to protect animals from PTZ-induced convulsion, which is serving as a guide for the study of anticonvulsant activity.

### 2.4. Statistical Analysis

All results were expressed as mean ± SEM. Statistical significance between the groups was analyzed by one-way analysis of variance (Sigma Plot 14 (Sigma Plot Software, Inc., La Jolla, CA, USA) software) followed by Tukey's post hoc multiple comparison test for comparison between groups. For the tests, *p* < 0.05 was considered statistically significant.

### 2.5. Ethical Considerations

The protocol was approved by the Institutional Review Board, College of health sciences, Addis Ababa University (protocol number: 053/15/Physio). Before, during, and after the study, animals were handled according to OECD guidelines [15].

## 3. Results

### 3.1. Yields of Hydroethanolic Extract and Its Solvent Fraction of *Artemisia afra*

The yields of the crude extract and the solvent fractions of *A. afra* crude extract are shown in Tables 1 and 2, respectively.

### 3.2. Effect of the Crude Extract on the Onset of PTZ-Induced Convulsion

**Figure 1:**
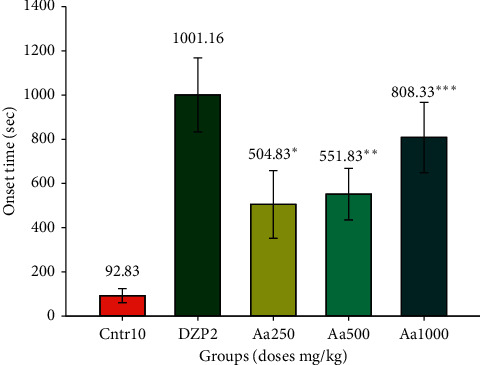
Mean onset of convulsions with hydroethanolic *A. afra* crude extract (250 mg/kg, 500 mg/kg, and 1000 mg/kg) 30 min before subcutaneous PTZ administration compared with those of the positive (diazepam 2 mg/kg) and negative (physiological saline 10 mg/kg) controls. Data represent mean ± SEM of three independent experiments (*n* = 6 for each group). SEM = standard error of the mean, Aa = *A. afra*, DZP = diazepam, and Ctrl = control.

The three doses (250, 500, and 1000 mg/kg) of crude extract of *A. afra* used showed a significant delay (504.833 ± 62.835^*∗*^ s; *p* < 0.05^*∗*^; 551.833 ± 47.69^*∗∗*^ s; *p* < 0.01^*∗∗*^; and 808.333 ± 64.8^*∗∗∗*^ s; *p* < 0.001^*∗∗∗*^, respectively), in the mean onset of PTZ-induced convulsion (*p* < 0.05,  *p* < 0.01,  *p* < 0.001, respectively), in a dose-dependent manner compared to the negative control (92.833 ± 13.006 s). The delay in the onset of convulsion was significantly less than that of diazepam (1001.167 ± 68.430 s) as shown in Figure 1.

### 3.3. Effect of the Crude Extract on the Duration of Convulsions

**Figure 2:**
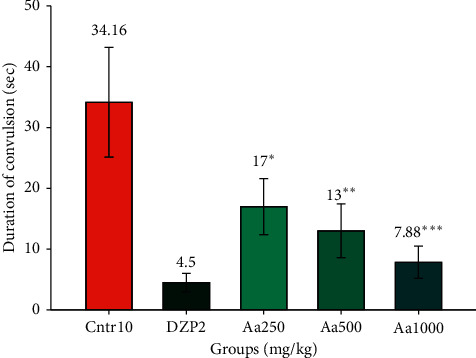
Mean duration of convulsions with hydroethanolic *A. afra* crude extract (250 mg/kg, 500 mg/kg, and 1000 mg/kg) 30 min before subcutaneous PTZ administration compared with those of the positive (diazepam 2 mg/kg) and negative (physiological saline 10 mg/kg) controls. Values are mean ± SEM of three independent experiments (*n* = 6 for each group). SEM = standard error of the mean, Aa = *Artemisia afra*, DZP = diazepam, and Ctrl = control.

The crude extract of *A. afra* significantly decreased the duration of PTZ-induced convulsions at 250, 500, and 1000 mg/kg doses (17.000 ± 1.88^*∗*^ s, *p* < 0.05^*∗*^; 13.000 ± 1.8^*∗∗*^ s, *p* < 0.01^*∗∗*^; and 7.833 ± 1.07^*∗∗∗*^ s, *p* < 0.001^*∗∗∗*^, respectively) against PTZ-induced convulsion compared to the negative control (34.167 ± 3.683 s) in a dose-dependent manner, but the duration was significantly less long than that of diazepam (4.500 ± 0.619 s) as shown in Figure 2.

### 3.4. Effect of the Solvent Fraction on the Onset of PTZ-Induced Convulsion

**Figure 3:**
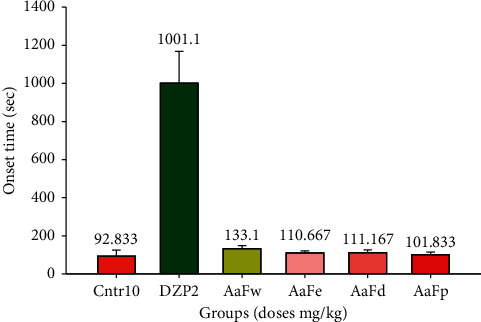
Mean onset of convulsion with 1000 mg/kg solvent fraction of hydroethanolic *A. afra* crude extract 30 min before subcutaneous PTZ administration for positive (diazepam 2 mg/kg) and negative (physiological saline 10 mg/kg) controls. Values are mean ± SEM of three independent experiments (*n* = 6 for each group). SEM = standard error of the mean, AaFw = *A. afra* water fraction, AaFe = *A. afra* ethanol fraction, AaFd = *A. afra* dichloromethane fraction, AaFp = *A.afra* petroleum ether fraction, DZP = diazepam, and Ctrl = control.

No significant difference was observed with all solvent fractions on the onset of PTZ-induced convulsion compared to the negative control, but the difference was significant than that of diazepam (*p* < 0.05) as indicated in Figure 3.

### 3.5. Effect of the Solvent Fraction on the Duration of Convulsion

**Figure 4:**
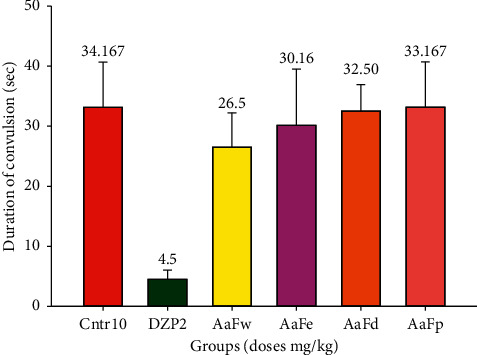
Mean duration of convulsion with 1000 mg/kg solvent fraction of hydroethanolic *A. afra* crude extract 30 min before subcutaneous PTZ administration for the positive (diazepam 2 mg/kg) and negative (physiological saline 10 mg/kg) control groups (*n* = 6 for each group). Values represent mean ± SEM of three independent experiments (*n* = 6 for each group). SEM = standard error of the mean, AaFw = *A. afra* water fraction*, AaFe* = *A. afra* ethanol fraction, AaFd = *A. afra* dichloromethane fraction, AaFp = *A. afra* petroleum ether fraction, DZP = diazepam, and Ctrl = control.

The solvent fractions of *A. afra* did not show a significant difference on the duration of PTZ-induced convulsion compared to the negative control, but the difference was significant than that of diazepam (*p* < 0.05) as indicated in Figure 4.

## 4. Discussion

Pentylenetetrazol exerts its convulsant effects by inhibiting the activity of GABA at GABA_A_ receptors, which resulted in inhibition of GABA-ergic neurotransmission in the central nervous system [17, 18].

GABA-ergic neurotransmission is closely associated with the induction of epilepsy in animals. The enhancement of GABA neurotransmission attenuates convulsions, while inhibition of the neurotransmission of GABA enhances convulsions [19].

GABA is an endogenous agonist of the GABA_A_ receptor (ionotropic receptor) facilitating the opening of the channels to chloride ions in the neuronal membrane leading to hyperpolarization resulting in impeding action potential transmission and hence responsible for the antiepileptic effects of the drugs that directly bind and activate GABA_A_ receptors or influence GABA release, transport, and metabolism. GABA-mediated chloride channel GABA-benzodiazepine receptor complexes are closely associated with the induction and onset of seizures [18, 19].

PTZ also increases the free radicals in the brain that leads to neuronal damage-induced epilepsy [20]. It can be considered a model for human generalized and absence seizures [21, 22].

Diazepam, which generally inhibits sodium currents and potentiates GABA transmission or enhances GABA-mediated inhibition in the brain, is employed as a standard antiepileptic drug in experiments that target the search for new antiepileptic agents as it blocks PTZ-induced convulsions [23].

The present study showed that the hydroethanolic extract of *Artemisia afra,* delayed the onset and decreased the duration of PTZ-induced convulsions in a dose-dependent manner.

This finding is in agreement with other studies which also showed the anticonvulsant effects of pycnogenol extract [24], the aqueous extract of *Antiaris toxicaria* stem bark [25], hydroethanolic *Phoenix dactylifera* fruit extract, and *Pimpinella anisum* oil [26].

The ability of the extracts to delay the onset and/or shorten the duration of convulsions was considered an indication of anticonvulsant activity [25, 27].

As the crude extract prevented PTZ-induced convulsion, the mechanism for the anticonvulsant effect of the crude extract might be associated with the activity on GABA synapses which diazepam acts on as well [17, 28].

The medicinal value of the plants lies in some chemical constituents (phytochemicals) that produce a definite physiological function as an anticonvulsant agent.

The most important of the bioactive constituents of the extracts are alkaloids, tannins, flavonoids, and phenolic compounds [29, 30].

This is in agreement with studies in which alkaloids, flavonoids, terpenoids, saponins, and coumarins were found to enhance GABA transmission to exert their anticonvulsant effect. Therefore, these phytochemicals may be responsible for the anticonvulsant activity observed in this study [31–34].

In contrast to the anticonvulsant activity observed in the crude extract, none of the solvent fractions demonstrated a significant anticonvulsant effect. This might be due to the too low concentrations of phytochemicals that are present in each solvent fraction to produce the expected effect. This is so because partitioning of the phytochemicals of the crude extract in different solvents may reduce the concentration to be present in each fraction. It might also be due to the loss of some phytochemicals undergoing decomposition during the partitioning process in contrast to what has been observed in another study where the solvent fractionation of *Ebenus stellata* crude extract did not reduce convulsion [35].

Our finding on solvent fractions is, however, in agreement with that of a previous study [36]. The anticonvulsant effect observed only in the crude extract might be attributed to the synergistic and/or additive effects as well as the higher concentration of the active ingredients present [37].

## 5. Conclusion

In summary, the results presented here indicate that the hydroethanolic crude extract obtained from *Artemisia afra* significantly delayed the onset and prevented the severity of PTZ-induced convulsions, indicating its anticonvulsant activity. While, the solvent fractionation of the hydroethanolic extract of the plant did not induce such effects, demonstrating a combination of different biologically active components in the hydroethanolic extract of the plant.

However, further studies are needed using different animal models for understanding underlying mechanism(s), and the active principles involved in these effects need to be explored.

The observed anticonvulsant effects confirm and justify the ethnomedicinal use of the plant in the management of epilepsy, as well as preserving the environment and its biodiversity.

## Figures and Tables

**Table 1:** Yield of crude extract of hydroethanolic (70% ethanol) extracts of *A. afra.*

Plant material	The total yield of extract (g)	Percentage of yield (%)
Hydroethanolic extract of *A. afra*	96.08	12.01

**Table 2:** Yields of the solvent fraction of 30 gm of 70% ethanol crude extract of *A. afra.*

Plant material	Type of fraction	Weight of fraction obtained (g)	Percentage of yield (%)
*A. afra*	Petroleum ether fraction	2.32	11.6
	Dichloromethane fraction	3.78	18.9
	Ethanol fraction	4.85	24.25
	Aqueous residue	9.56	47.8

## Data Availability

The datasets utilized or potentially examined during the analysis are accessible from the corresponding author and coauthors on demand, besides included in the supplementary materials file.

## Supplementary Materials

Supplementary Materials The datasets utilized or potentially examined during the analysis are included in the supplementary materials file.
