# Supplementary material for: Effect of 70% Ethanol Extract and its Solvent Fractions of Artemisia afra (Jacq. Ex Willd.) against Pentylenetetrazole-Induced Seizure in Mice
Source: Evid Based Complement Alternat Med. 2021 Jun 17;2021:6690965. doi: 10.1155/2021/6690965 (PMC8233086; doi:10.1155/2021/6690965)
Supplement: Supplementary Materials — The datasets utilized or potentially examined during the analysis are included in the supplementary materials file. [file 6690965.f1.docx]

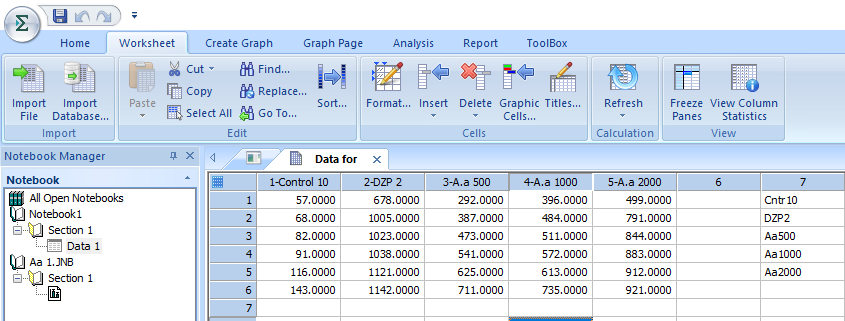


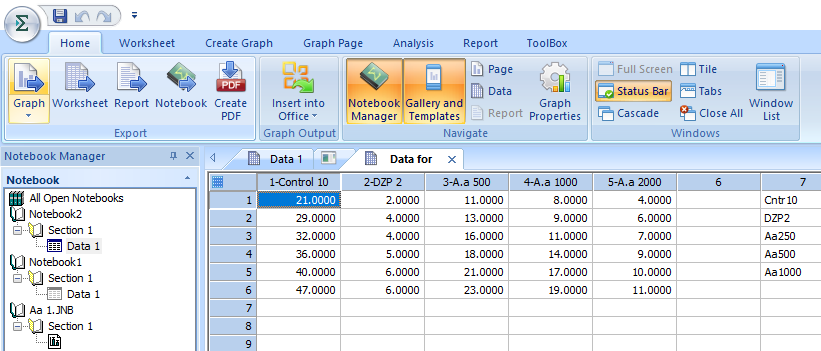


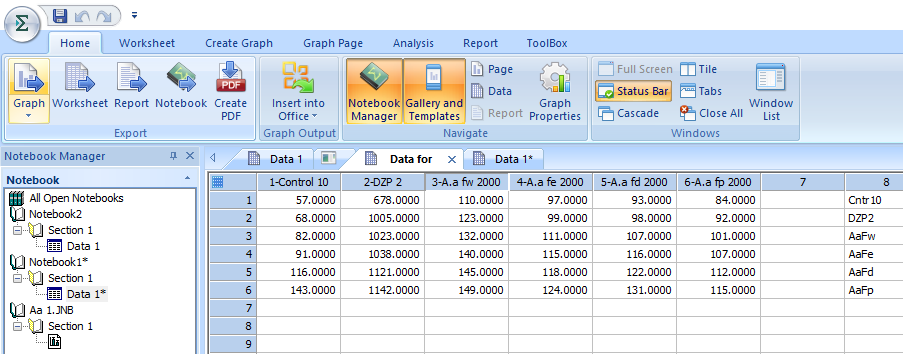


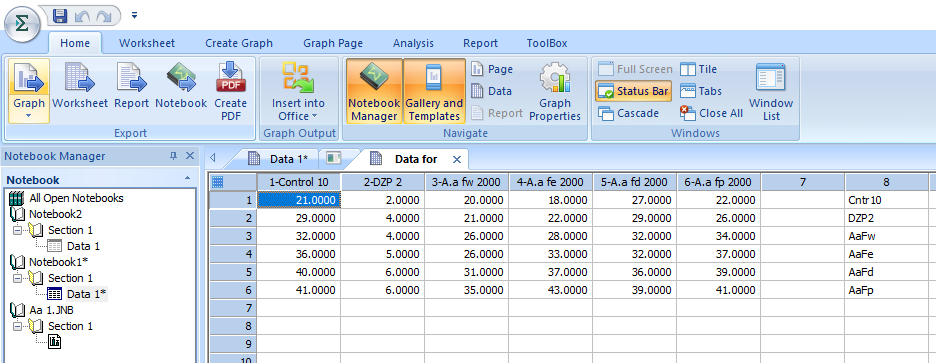


Description:

The datasets utilized or potentially examined during the analysis included in the supplementary materials file.
